# Detection of bovine serum albumin using hybrid TiO_2_ + graphene oxide based Bio – resistive random access memory device

**DOI:** 10.1038/s41598-019-52522-w

**Published:** 2019-11-06

**Authors:** Dwipak Prasad Sahu, S. Narayana Jammalamadaka

**Affiliations:** Magnetic Materials and Device Physics Laboratory, Department of Physics, Indian Institute of Technology Hyderabad, Hyderabad, 502 285 India

**Keywords:** Electronic devices, Electronic properties and devices

## Abstract

Bio – molecules detection and their quantification with a high precision is essential in modern era of medical diagnostics. In this context, the memristor device which can change its resistance state is a promising technique to sense the bio - molecules. In this work, detection of the Bovine Serum Albumin (BSA) protein using resistive switching memristors based on TiO_2_ and TiO_2_ + graphene oxide (GO) is explored. The sensitivity of BSA detection is found to be 4 mg/mL. Both the devices show an excellent bipolar resistive switching with an on/off ratio of 73 and 100 respectively, which essentially demonstrates that the device with GO, distinguishes the resistance states with a high precision. The enhanced performance in the GO inserted device (~ 650 cycles) is attributed to the prevention of multi-dimensional and random growth of conductive paths.

## Introduction

Indeed, resistive switching (RS) memristive devices and bio – resistive random access memory (RRAM) devices^1,2^ have the potential to replace the landscape of existing electronic devices pertinent to memory^3^, logic^4^ and sensing^5^ applications. Essentially, the functionality of memristive device can be attributed to a state of alteration and dynamics of the same can be engineered to a target specific application. Particularly when it comes for sensing, there has been a great interest in nano bio – sensing due to the possibility for minimal invasive, therapy personalization and real time monitoring.

Among various memristive devices^6–8^, RS memory device has gained much interest owing to its simple structure, easy fabrication, high density, excellent stability and low power consumption^9–11^. The responsible physical phenomenon that governs in memristive RS devices has been believed as migration of oxygen ions which modifies the resistive properties of the devices in a non – volatile manner. In general RS device consist two-terminals and the active material (typically transition metal oxide) would be sandwiched between two metal electrodes. Resistance of such a device can be switched between high resistance state (HRS) and low resistance state (LRS) by applying an electric field across the electrodes. If positive and negative polarities are mandatory to have SET (HRS to LRS) and RESET (LRS to HRS) switching, such RS can be termed as bipolar resistive switching (BRS). On the other hand, in unipolar RS (URS), one polarity is sufficient for SET and RESET switching. These memristive devices have many advanced applications such as neuromorphic systems^12,13^, memory logic^14,15^ and analogue computing^16^. Bio molecules such as proteins, DNA, enzymes, bioactive small molecules, dopamine and uric acid have been detected using nanomaterials like gold nanoparticles^17,18^, graphene^19,20^, carbon nanotubes^21,22^, nanowires^23^ and quantum dots^24^.

Among all the available proteins, bovine serum albumin (BSA) has been identified as multifunctional one and plays an important role in delivering the fatty acid/amino acid *etc*. Essentially, BSA can interact with many organic, inorganic molecules, drugs, ionic metals and radicals due to multiple binding sites on exposed surface of molecule apart from its biomedical application such as targeted drug delivery^25,26^. On top of that it consists of ample biochemical applications including enzyme – linked immunosorbent assay, immunoblots, and immunohistochemistry. It has been believed that reduction of BSA may cause various diseases in cattle families. In this respect, the quantification of BSA is very much crucial in order to understand the severity of particular disease. Some methods have been explored to detect the BSA such as triangular silver nanoplates using spectrophotometric method^27^, optical fiber based on a Mach-Zehnder interferometer *etc*^28^. However, these state-of-the-art technologies of detecting BSA protein by optical methods are cumbersome, labor intensive, which demands an alternative approach with environmental friendly, non-toxic and easy detection method. Hence, it is important to explore detection of BSA using TiO_2_ based RS memristor due to its low operational voltage and with high sensitivity^29^. Upon adding BSA protein to TiO_2_ RS memristor device, multiple binding sites of BSA (R-COO− and R-NH^+^_3_) may interact with predominant surface groups of TiO_2_ (Ti_2_ = O−, Ti-OH, Ti_2_ = OH), which may lead to drastic change in transport/optical properties of the TiO_2_ memristor device. On top of that it has been proved that graphene oxide (GO) consists of oxygen containing functional groups^30^. As a result of this, the conductivity/optical properties of the device may enhance by introducing GO as a sandwich layer between BSA and TiO_2_. Salient features of present work are (a) detection and confirmation of BSA using TiO_2_ and TiO_2_ + GO based RS memristor devices (b) increased sensitivity in a device with GO (c) excellent on/off ratio ~100 (d) achieving multi-bit data storage by controlling the compliance current (e) confirmation of filamentary kind of switching using conductive surface atomic force microscope (C – AFM).

## Results and Discussion

**Figure 1:**
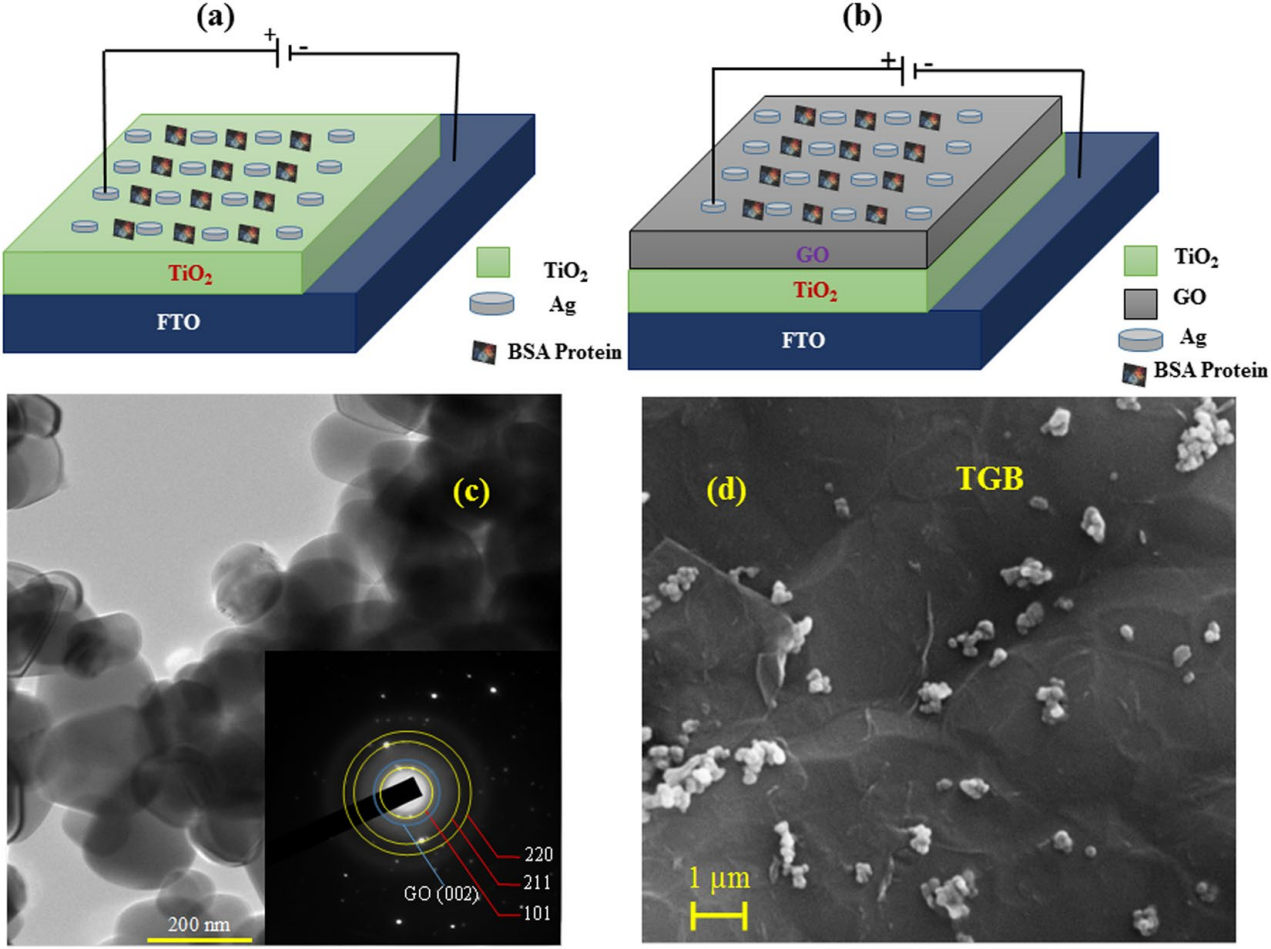
Schematic of the device on which I–V measurements were performed using two probe method with a Keithley 2400 Source meter. **(a)** Consists of the configuration as Ag/BSA/TiO_2_/FTO (TB). Here, fluorine doped tin oxide is used as bottom electrode and Ag as top electrode. Initially TiO_2_ is deposited on top of FTO and then BSA protein was added to it. Current perpendicular to the plane configuration was used to perform I–V characteristics by keeping electrical probes one on FTO and other one on Ag pad. **(b)** To enhance the detection sensitivity of low concentration of BSA, a GO layer was introduced between TiO_2_ and BSA due to which device configuration was changed to Ag/BSA/GO/TiO_2_/FTO (TGB). **(c)** TEM image of TiO_2_ + GO nanoparticles measuring about 120 nm. Inset shows the selected area diffraction pattern indicating anatase phase of TiO_2_ and presence of GO (002) plane in the complex. **(d)** The morphological features of BSA molecules after encapsulation on TiO_2_ + GO surface were characterized by Field Electron Scanning Electron Microscopy (FESEM) at a voltage of 20 keV which reveals the spherical particle like morphology distributed on the surface of graphene oxide.

Figure 1a,b depict schematic of the devices that we used to perform I–V characteristics. Figure 1a consists the configuration as Ag/BSA/TiO_2_/FTO (TB). Here, fluorine doped tin oxide (FTO) was used as bottom electrode and Ag as top electrode. Initially TiO_2_ was deposited on top of FTO substrate by drop casting method and subsequently BSA was added on TiO_2_ for the possible detection of it. The concentration of BSA was varied from 0–15 mg/mL (4 mg/mL, 6 mg/mL, 8 mg/mL and 15 mg/mL respectively). I–V characteristics were carried out using two probe method by Keithley 2400 source/sense meter. In all the devices that we discuss in this paper, the current was sent perpendicular to the plane of the film (CPP) configuration. Figure 1b depicts the device with configuration Ag/BSA/GO/TiO_2_/FTO (TGB). The importance of graphene oxide layer (GO) is to enhance the detection sensitivity of the BSA with lower concentration. Results those demonstrated in this manuscript are reproducible and also tested with various devices.

Figure 1c shows the TEM image of polycrystalline TiO_2_ nanoparticles + GO with an average particle size of 120 nm. Polycrystalline nature of TiO_2_ was confirmed by selected area diffraction pattern (SAED) as shown in the inset of Fig. 1c. The diffraction rings were indexed to the anatase TiO_2_ structure (yellow). The presence of (002) plane (blue) indicates the presence of GO in the composite. Figure 1d shows the Field emission scanning electron microscope (FESEM) image of BSA/GO/TiO_2_/FTO device. The voltage applied was 20 keV. It is clear from the graph that BSA molecules are present on the top surface of the device. The preliminary characterization of the BSA protein and the presence of protein in TGB compound were confirmed by UV-Vis spectroscopy and FTIR measurement (Fig. S1 in the Supporting Information).

For albumin protein detection various analytical methods have been developed, however, spectrophotometric methods are fast and highly sensitive. Essentially, upon binding the protein with oxide surface, there would indeed be a change in the optical properties of resultant composite (TiO_2_ + BSA) and the same can be confirmed with the spectroscopic methods. In the similar direction, spectroscopic methods like absorbance and fluorescence methods have been envisaged to detect DNA concentration, DNA damage, glucose, detection of rabbit IgG using TiO_2_ nanoparticles^31–34^. Zhang *et al*. has demonstrated a simple and highly sensitive method to detect BSA protein using triangular silver nanoplates using UV-Vis spectrophotometry^27^. Yet in another study, gold nanorods have been used to detect the concentration of albumin using both the absorbance and fluorescence methods^35^. In the present case, we used both the methods to confirm the presence of BSA. The spectroscopic method of BSA detection is shown in the supporting information.

**Figure 2:**
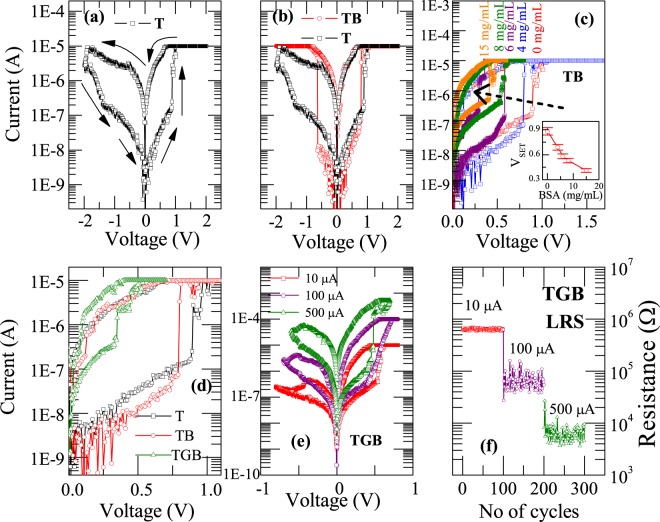
I–V characteristics of **(a)** Ag/BSA/TiO_2_/FTO and **(b)** Ag/BSA/GO/TiO_2_/FTO devices in voltage sweeping mode (with a compliance current of 10 µA) at room temperature showing bipolar switching. The detection of BSA protein is manifested as a shift in values of V_SET_ from 0.9 V (for T) to 0.77 V (for TB). **(c)** Effect of protein on the switching voltages were studied on TiO_2_ by varying the concentration of protein which indicates a systematic shift of V_SET_ for different concentration of BSA. Inset shows variation of V_SET_ with BSA concentration. **(d)** Enhancement in the switching performance has been observed by inserting a graphene oxide layer between TiO_2_ and BSA protein with V_SET_ changes from 0.77 V (TB) to 0.32 V (TGB). The systematic shift of V_SET_ can be seen for all three devices (T, TB, TGB). **(e)** Multi-bit data storage of TGB device under different compliance currents of 10 µA, 100 µA, 500 µA. **(f)** Endurance characteristics of TGB device in LRS state for different compliance current at 10 mV read voltage.

Figures 2a,b represents the evidenced I–V characteristics of Ag/TiO_2_/FTO and Ag/BSA/TiO_2_/FTO devices respectively. Initially the device Ag/TiO_2_/FTO is at high resistance state and upon sweeping voltage from 0–2 V, indeed there is a sharp jump in current at 0.9 V, which indicates that device changes its resistance state from HRS to LRS and the corresponding voltage can be termed as V_SET_. Above V_SET_, as there is a compliance limit of 10 μA (to avoid dielectric breakdown), we do see a saturation for the current that we measured. In the reverse run from 2 V–−2 V, the device is in LRS until −1.9 V (V_RESET_). Above this again it reaches to HRS and continued to be in the same state until 0 V. Such a behavior (having V_SET_ and V_RESET_ for different polarities of the voltage) is typical for a bipolar resistive switching (BRS). In the present investigation, we have observed BRS on all the devices that we investigated. Bipolar resistive switching has well been established in TiO_2_ based resistive random access memory devices^36,37^.

Now we discuss about detection of BSA protein with TiO_2_ based Bio – RRAM through electrical measurements. In order to demonstrate our efforts, initially we introduced BSA protein on top of TiO_2_ layer and investigated the resistive switching properties. The detection of BSA protein is manifested as a shift in values of V_SET_. Figure 2b manifests such a shift in V_SET_ upon adding BSA. V_SET_ is shifted from 0.9 V (for T) to 0.77 V (for TB) and this variation of V_SET_ with BSA addition motivated us to perform a systematic study. Pertinent to this study, we added different concentrations of BSA (4, 6, 8 and 15 mg/mL) on independent device and looked into resistive switching properties. Figure 2c shows the systematic shift of V_SET_ for different concentration of BSA. For instance, the value of V_SET_ changes from 0.9 V (for 0 mg/mL) to 0.4 V (for 15 mg/mL), hinting that the present device is highly sensitive for the detection of BSA protein. In order to make it more clear, we also showed the variation of V_SET_ with BSA concentration as an inset of Fig. 2c. Previous studies on transport properties of single layer BSA protein in molecular transistors has exhibited a low operational voltage^38^. In addition, a two terminal transport experiment at nano-scale has shown Ohmic behavior, which indicates the metallic nature of the protein^39^ and leads to a decrease in switching voltage with an increase in the BSA concentration. It could be seen that the HRS current increases by 2 orders of magnitude when protein concentration enhances to 15 mg/mL. The lower value of resistance in HRS could be due to electrons transfer between protein molecules, which may be due to decrease in average separation between protein molecule upon increasing the concentration. We also compared the sensitivity of our device with previous literature reports^27,35^ where they could able to detect as low as 0.5 ng/mL and 0.2 mg/mL (UV region), 0.05 mg/mL (Vis-IR region), 0.0013 mg/mL (by Fluorescence spectra) respectively. However, in our case we could able to detect 4 mg/mL by electrical measurements.

In order to improve the sensitivity of BSA protein detection, we introduced a GO layer between TiO_2_ and BSA protein. Corresponding results are shown in Fig. 2d. It is evident that for 4 mg/mL of BSA protein, the V_SET_ changes from 0.77 V (TB) to 0.32 V (TGB), which suggests that the GO indeed enhances the transport properties by pushing V_SET_ to a lower value. This is an important observation which may have many practical implications. It could be that GO layer provides better environment for conductive filament growth inside the switching layer, which offer lower switching voltages and good endurance characteristics. As a result of above, the randomness of filament evolution is prevented by inserting a GO layer and only a fewer conductive paths are stabilized for the switching. During set process, we believe that oxygen groups are removed from GO, which may migrate towards bottom electrode. Due to the removal of oxygen group, the C–C bonds on GO may shorten, which leads to shorter hopping distance for the carriers. Essentially, shorter hopping distance makes easy electron migration and can accompany lower resistance and lower values of voltages for switching the device^40^.

Multibit data storage capability of the device is checked with different values of compliance current say 10 µA, 100 µA and 500 µA. It is evident from Fig. 2e that three different LRS states are distinguishable for different compliance limits. In addition, higher the compliance limit, better is the LRS current and stronger the filament. Figure 2f depicts the evidenced LRS state endurance behavior of TGB device with different compliance current limit of 10 µA, 100 µA and 500 µA and at a readout voltage of 10 mV. It is clear from both the experiments that we could tune the LRS states with different compliance limits. On top of that, reading of various resistance states are successfully achieved at 10 mV, which demonstrates that compliance current is a key parameter and can allow one to access multilevel LRS states.

Now we discuss about the conceivable mechanism for the BSA protein adsorption on TiO_2_ surface. It has widely been investigated by many researchers and reported a conformational change in the protein molecule due to adsorptions on TiO_2_ surface^41,42^. This change not only influences the characteristics of adsorbed protein but also directs the behavior of surface. In our experiment as we used DI water, the pH of it is around 7.4. In this pH region the BSA protein and TiO_2_ surfaces can carry negative sign and can have electrostatic repulsion, which leads to less adsorption onto oxide surface^43^. Whatever may be the case, the absorption of protein on oxide surfaces can primarily be happen through (a) electrostatic attraction and (b) ligand exchange between BSA and oxide surfaces. The adsorption of protein on oxide surfaces can happen by van der Waals forces, the sorbate conformational stability and hydrophobic interactions. Surface hydrophilicity, surface charge and aggregation of oxide particles can also provide the possible spaces for protein adsorption on oxide surfaces^44,45^. As electrostatic interactions is one of the main driving forces in protein adsorption, although the overall charges of both TiO_2_ and protein surfaces have same sign, adsorption can still happen through electrostatic attraction. The conceivable mechanism might be that different regions of protein surface consists different signs of charge (positive or negative), which can attract positively charged particle surfaces^46^. As there is less adsorption, the excess negative charges may participate in the switching process which causes the devices to form filaments with a lower set voltage.

If we consider an experiment with condition of pH 7.4, the predominant surface groups of TiO_2_ are Ti_2_ = O− and Ti-OH, with few Ti_2_ = OH. On the other hand, the important functional group of BSA protein are R-COO− and R-NH_3_+^43,47^. We believe that an interaction between these functional groups lead to surface modification and predominant change in I–V curves with a shift in switching voltages. The kind of electrostatic interaction that occurs between aforementioned groups are given below

1 $${\rm{Ti}}-{\rm{OH}}+{\rm{R}}-{{\rm{NH}}}_{3}^{+}+\to {\rm{Ti}}-{{\rm{OH}}}_{2}^{+}:{{\rm{NH}}}_{2}-{\rm{R}}\,({\rm{electrostatic}}\,{\rm{interactions}})$$

2 $${\rm{Ti}}-{\rm{OH}}+{\rm{R}}-{{\rm{COO}}}^{-}+\to {\rm{Ti}}-{\rm{OH}}\ldots {}^{-}{\rm{O}}{\rm{OC}}-{\rm{R}}\,({\rm{electrostatic}}\,{\rm{interactions}})$$

3 $${{\rm{Ti}}}_{2}={{\rm{O}}}^{-}+{\rm{R}}-{{\rm{NH}}}_{3}^{+}\to {{\rm{Ti}}}_{2}={{\rm{O}}}^{-}{}^{+}{\rm{N}}{{\rm{H}}}_{3}-{\rm{R}}\,({\rm{hydrogen}}\,{\rm{bonding}}\,{\rm{interaction}})$$

**Figure 3:**
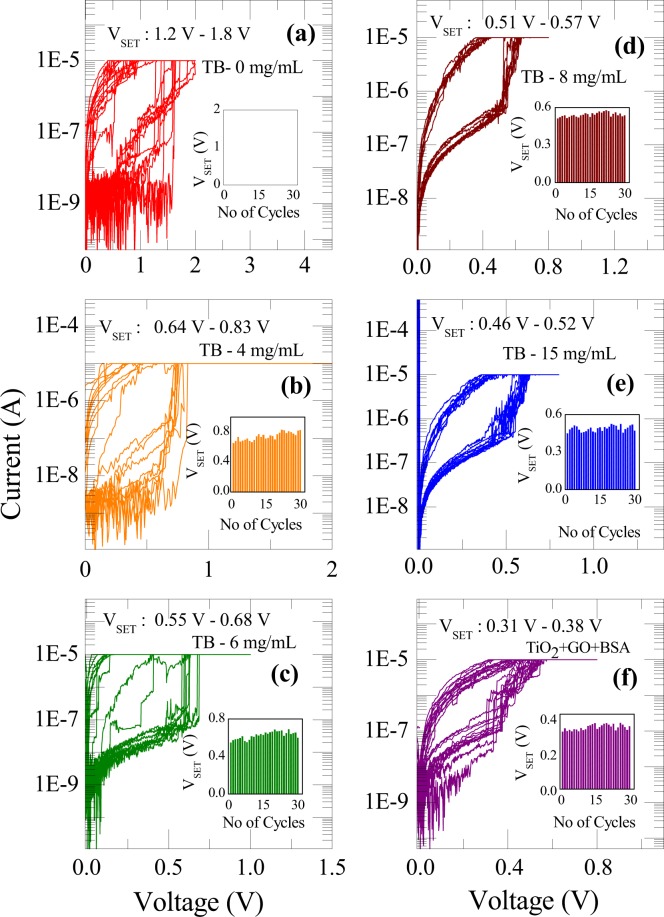
Resistive switching of TiO_2_ based RRAM devices with different concentrations of BSA. These graphs differentiate real signal change to noise range for I-V detection of the BSA with different concentrations. Inset of each graph shows that there is no significant change in switching voltage of the devices with number of cycles.

In order to differentiate real change in signal to noise for these devices, we have established the noise range for I-V detection of BSA with different concentrations. Figure 3(a–f) shows such noise measurements, which demonstrates range of voltages (noise) at which SET switching can happen for different concentrations of BSA protein. Upon repeated voltage cycling, we do not see a predominant variation in switching voltages, which demonstrates the stability of the filaments. For instance, the noise for change in voltage for parent device is ~0.6 V. However, the range of noise for voltage diminishes to 0.06 V for BSA with concentration (15 mg/mL). This indeed, demonstrates the enhanced stability of filament in all the devices with BSA. It is apparent from the figure that each device with specific concentration has its own noise range for voltage. Inset of each graph shows that there is no significant change in switching voltage for the devices despite repeated cycling.

Now we discuss about the actual mechanism for the detection of BSA through our XPS measurements. The actual thought to prove our claim is that the amount of voltage that one need to apply to get switching would essentially depends on the number of oxygen vacancies. For instance if a film has more oxygen vacancies, less would be the voltage required for the filament formation and SET switching. On the other hand, if a film consists less oxygen vacancies, more would be the voltage required for the filament formation and SET switching. Hence, we believe that number of oxygen vacancies would decide whether the switching must happen at lower voltages or higher voltages.

**Figure 4:**
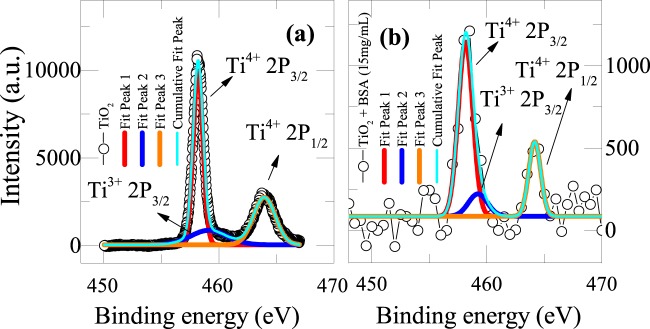
**(a)** XPS spectra of TiO_2_ indicating the two main peaks of Ti^4+^; Ti^4+^ 2p_3/2_ (binding energy of 458.1 eV), Ti^4+^2p_1/2_ (binding energy of 463.9 eV) and a shoulder peak at binding energy of 459.1 eV corresponding to Ti^3+^ 2p_1/2_ in Ti_2_O_3_. **(b)** XPS data of 15 mg/mL added BSA protein (TiO_2_ + BSA).

Particularly in TiO_2_ based RRAM devices, oxygen vacancies are the primary constituents for the filament formation. Upon adding, BSA to TiO_2_ device, we could able to see that there is decrease in the switching voltage, which may be due to the enhanced number of oxygen vacancies. We believe that the chemical reaction between predominant surface groups of TiO_2_ (such as Ti_2_ = O^−^ AND Ti-OH, Ti_2_=OH) and functional groups of BSA protein (R-COO- and R – NH3+) might be playing an important role in controlling number of oxygen defects in the device. In order to confirm our claims, we have performed X-ray photoelectron spectroscopy (XPS) measurement on the parent device TiO_2_ (Fig. 4(a)) and TiO_2_ + BSA (15 mg/mL) (Fig. 4(b). It is evident from Fig. 4(a) that the Ti^4+^ peak exists in doublet state and corresponding peaks are related to Ti 2p_3/2_ (binding energy of 458.1 eV) and Ti 2p_1/2_ (binding energy of 463.9 eV), which is consistent with the earlier reports. In addition to this, there is a broad peak at binding energy of 459.1 eV corresponding to Ti^3+^ 2p_1/2_ in Ti_2_O_3_^48,49^. Increased number of oxygen vacancies are identified directly from the area under the peak of Ti^4+^. Essentially, the area under the curve of Ti^4+^ peak diminishes upon adding BSA concentration of 15 mg/mL (Fig. 4(b)). The reduction of Ti^4+^ peak area in the 15 mg/mL added BSA protein (TiO_2_ + BSA) compared to parent TiO_2_ indicates the generation of oxygen vacancies to compensate and balance the system electrostatically. Corresponding chemical equation can be written as follows^50^.

$$4{{\rm{Ti}}}^{4+}+{{\rm{O}}}^{2-}\to 4{{\rm{Ti}}}^{4+}+2{{\rm{e}}}^{-}/\square \,+\,{}_{2}{}^{1}{\rm{O}}_{2}\to 2{{\rm{Ti}}}^{4+}+2{{\rm{Ti}}}^{3+}+\,\square +\,{}_{2}{}^{1}{\rm{O}}_{2}$$

where □ is an empty site originated due to the removal of O^2−^ in the lattice.

Thus, it is expected that the concentration of oxygen vacancies might be increasing with increasing the protein concentrations in the TiO_2_ based RRAM devices, which actually helps in detecting the BSA protein by indicating a reduced switching voltage in the I–V characteristics of the device. S. carrara *et al*. has also proposed detection of rabbit antibodies through I–V characteristics with silicon nanowire memristive bio-sensors where they have pointed out a voltage gap in the bio-functionalized device and the same has been attributed as a detection parameter^1^. We also believe that the BSA protein might be encapsulating the TiO_2_ particles. This might be the reason why we do see lot of noise in the XPS data with BSA (Fig. 4(b)).

**Figure 5:**
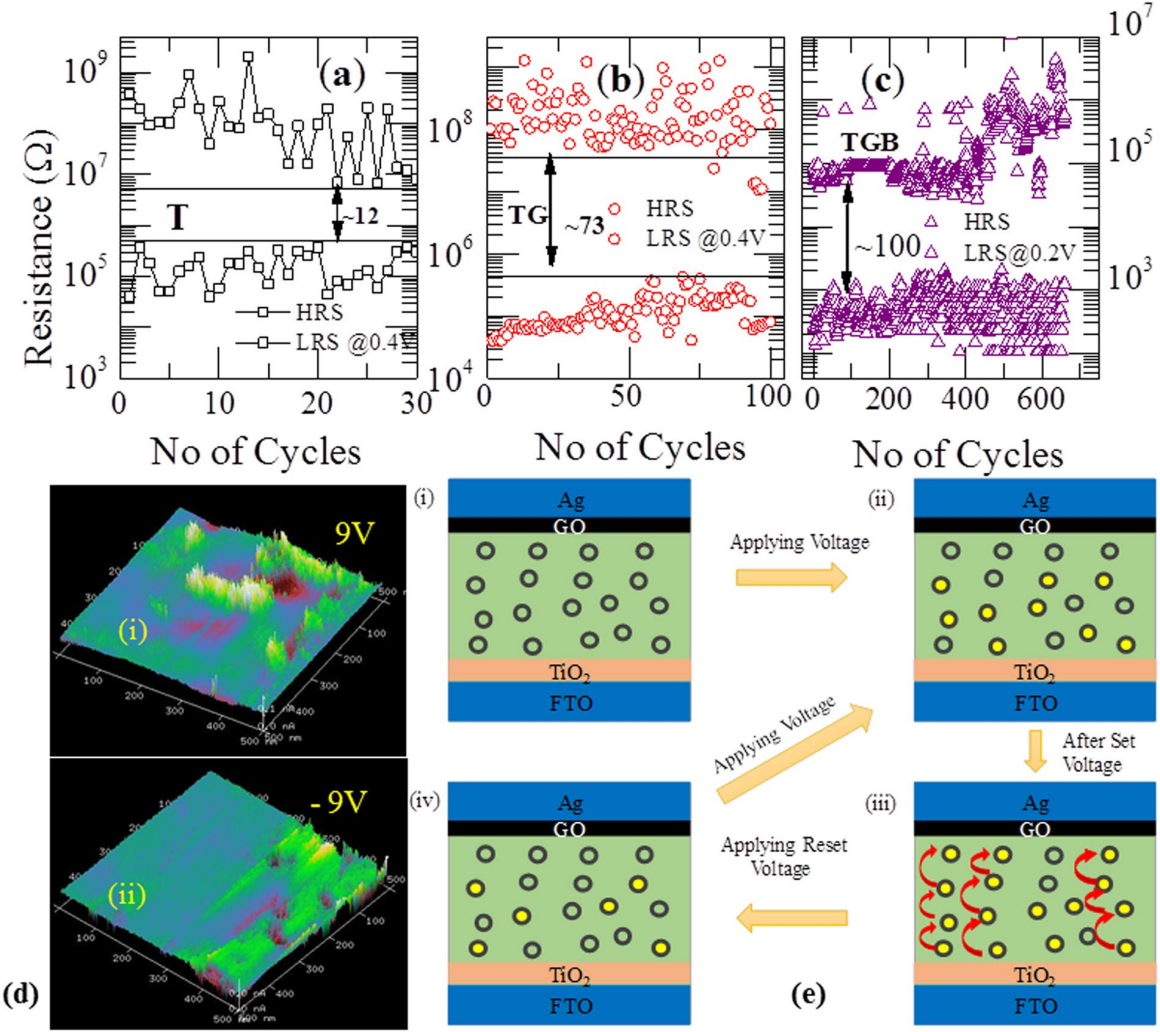
Endurance performance of the three devices. **(a)** Ag/TiO_2_/FTO based RRAM device was repeatable for 30 cycles with a on/off ratio of 12 **(b)** On/Off ratio increases to 73 for the Ag/BSA/TiO_2_/FTO device **(c)** graphene oxide inserted device (TGB) shows enhanced endurance characterists and increase in the resistance ratio up to 10^2^ at 0.2 V read voltage. **(d)** Current 3D conducting atomic force microscope (C-AFM) images of the Ag/BSA/GO/TiO_2_/FTO device scanned in an area of 1 × 1 µm^2^ by applying a voltage bias of 9 V and −9 V respectively. (i) Formation of conductive channels on applying a bias voltage of 9 V (ii) disappearance of conductive paths in the reverse bias of −9 V indicating the role of nano-channels in the switching phenomena. **(e)** Schematic representation of the switching mechanism in the Ag/BSA/GO/TiO_2_/FTO device, where the hollow circles represent empty charge trapping sites and the solid circles depict the filled charge sites. (i) Pristine state of the device without external bias. (ii) When a positive potential is applied to the top electrode, charge carriers injected from electrode fill few of the empty charge sites (iii) As the bias voltage reaches set voltage, most of the charge trapping centers are filled and hopping of charges from one filled sites to other is initiated, resulting in the formation of conductive paths which corresponds to the low resistance state. (iv) When reset voltage is reached, dissolution of conductive filaments occurs in the weakest part of the filament and the device switches to HRS.

Now we discuss about endurance characteristics of the device. Cycling endurance of RRAM device is one of the important features to be studied from the application point of view. Memory margin of all RRAM devices are calculated using the formula (R_OFF_  −  R_ON_)/R_ON_ ≈ R_OFF_/R_ON_. In general, a memory window of 10 is sufficient enough to distinguish information between HRS and LRS. As shown in the Fig. 5a, TiO_2_ based RRAM device was repeatable for 30 cycles with resistance ratio of 12. However, with addition of BSA protein, the ratio increased to 73 and the device can be switched for 100 cycles (Fig. 5b). It is evident from the Fig. 5c that TGB device shows more durability and up to 650 cycles we could switch between ON/OFF with a ratio of 100 after which the device fails. In oxide based RRAM devices, the most common defects are oxygen vacancies. Thus, when an external bias is applied to the active material, oxygen ions are pulled out of the equilibrium position, which results in the generation of vacancies and current conduction. This is believed to happen in the devices through oxygen vacancies based on conductive filaments  between top and bottom electrodes. Similarly, the filaments rupture due to drifting of oxygen ions from top metal electrode and recombining with the vacancies. However, as the number of switching cycles increase, additional defects are generated in the vicinity of filaments which results in an increase of the radius of filaments^51^. Moreover, the different electrode material in the device structure is very likely to form a wider filament on one side and a narrower in other side. Due to continuous accumulation of vacancies, the recombination rate of oxygen ion and vacancy pairs are also reduced, which eventually leads to device failure^52^. Therefore, the possible reason for the device failure after 650 cycles could be due to oxidation of metal electrode or an abrupt increase in the local density of oxygen vacancies and formation of additional conductive channels in the active layer which have resulted in lowering the resistance of the device and remains in an irreversible LRS state, which indicates the endurance failure.

Conductive Atomic Force Microscopy (C-AFM) is a powerful technique  and is used  to map conductive channels. This has the ability of spatially resolving local heterogeneities of electrical response and simultaneously probing the current distribution on the surface of the material. In this study, the metallic AFM tip behaves as a nano-sized electrode in the switching of the device. Images were taken using Bruker SCANASYST-AIR Probes (Silicon Tip on Nitride Lever) having 12 nm, 70 kHz and 0.4 N/m as curvature radius, oscillation frequency and cantilever spring constant respectively. The experiment was carried out at room temperature and at atmospheric pressure without applying any current compliance. Figure 5d illustrates the current distribution C-AFM image of TGB based memory device. We applied a +/−9 V bias on an area of 1.0 × 1.0 µm^2^ at 1.01 Hz in contact mode. The current mapping images were obtained by moving the cantilever across the film surface. When a positive potential was applied, a large number of conductive filaments are seen on the surface and is shown in Fig. 5d(i). On the other hand, filaments are not evident for the opposite bias voltage and are shown in Fig. 5d(ii). Hence, the presence of conductive paths are due to widely accepted filament model resistive switching.

The variation of HRS and LRS is explained on the basis of Schottky emission mechanism (ln I vs V^1/2^ variation is linear) and trap controlled space charge limited conduction mechanism (consists of ohmic, trap assisted region and space charge region). More details about the behavior are given in supporting information. Conceivable physical mechanism for both the mechanisms can be explained as follows using Fig. 5e. In TiO_2_, the most common defects are oxygen vacancies and these oxygen vacancies are believed to form trap sites for electrons^53,54^. When no external bias is applied, the device would be in HRS state as shown in Fig. 5e(i). At low voltages, some trap centers would be filled with charge carriers and at high voltage, hopping of charge carriers would takes place through charge trapping centers to Ag electrode, which leads to low resistance state for the device (Fig. 5e(ii,iii)). HRS state can be achieved by applying reset voltage, due to the fact that injected charge carriers would break the weakly linked conduction in the device (Fig. 5e(iv))^55,56^.

## Experimental Section

### Material preparation

#### Synthesis of graphene oxide

For the preparation of GO, modified Hummer’s method^57^ was adapted. First, the precursor solution was prepared by mixing 1 g of graphite powder and 0.5 g of sodium nitrate to 23 ml of concentrated sulfuric acid. Then, 3 g of potassium permanganate was added slowly to the prepared solution after 1 hr. To prevent thermal explosion, the solution beaker was kept in ice bath and stirred constantly for 12 hours at 35 °C. Further, this solution was diluted by adding 500 ml of deionized water under vigorous stirring and then treated with 30% H_2_O_2_ (5 ml). Finally, the mixture was washed with hydrochloric acid and deionized (DI) water and GO was obtained by filtering and drying the resultant material.

On top of that TiO_2_ (powder form) and BSA proteins were purchased from Sigma-Aldrich and Himedia respectively. Subsequently, solution of BSA was prepared by dissolving 50 mg of BSA to 10 ml of deionized water. In addition, both the TiO_2_ and GO solutions were prepared by adding them to DI water with 1 mg/ml respectively.

### Device fabrication

Using the above prepared solutions, we have fabricated three resistive random access memory (RRAM) devices, Ag/TiO_2_/FTO (T), Ag/BSA/TiO_2_/FTO (TB), Ag/BSA/GO/TiO_2_/FTO (TGB) respectively. Prior to device fabrication, ultrasonication was used to clean FTO substrates. Cleaning of FTO substrate was done at least 10 min with deionized water, acetone and 2 – propanol individually. For all devices, FTO was used as bottom electrode and silver as top electrode. The resistance of FTO is approximately 15 Ω. The devices were prepared by drop casting the solution onto FTO substrate and dried at a temperature of 75 °C (for T device). A relatively low temperature of 35 °C was supplied to TB and TGB devices for the drying purpose in order to prevent the damage for BSA proteins if any. Silver conducting epoxy was used as top electrode. The UV-Vis absorbance spectra of BSA protein, all three devices (T, TB, TGB) were recorded using a Perkin Elmer LAMBDA spectrophotometer between 200 to 700 nm. Chemical bonding within the protein was studied by Fourier transform infrared spectroscopy (FTIR), using a FTIR spectrometer Bruker Tensor 37 by ATR method in the range of 4000 to 500 cm^−1^ with a spectral resolution of 4 cm^−1^. The fluorescence (FL) spectra were collected by Edinburgh FLS 1000 fluorescence spectrometer with an excitation wavelength of 340 nm. Microstructural studies were done using the field emission scanning electron microscope (Zeiss ultra 55 FE-SEM). The transmission electron microscopy (TEM) investigations were performed using JEOL JEM 2100 instrument at an accelerating voltage of 200 kV. In order to prepare sample for TEM, the TiO_2_ and GO were dissolved in DI water and subsequently ultrasonicated for 10 minutes. The suspensions were dropped on to a standard carbon grid. The electrical measurements were performed at room temperature using a Keithley 2400 source meter with a compliance limit of 10 µA. In order to gain information on local electrical conduction and to observe filament formation, we have used a conductive atomic force microscope (C-AFM). In this method, nano-sized AFM tip itself works as top electrode. The measurement was performed by applying a voltage between the C-AFM tip which acts as top electrode and FTO as bottom electrode. We applied two different bias voltages, 9 V and −9 V, on an area of 1.0 × 1.0 µm^2^ at 1.01 Hz in contact mode.

### Summary

In summary, we have investigated the detection of BSA protein using TiO_2_ and TiO_2_ + GO based RRAM devices. Lowering of switching voltages, enhanced endurance characteristics (up to 650 cycles) and enhanced ON/OFF resistance ratios (~10^2^) were observed in TiO_2_ + GO RRAM device in comparison with bare TiO_2_ RRAM device. Enhanced characteristics in TiO_2_ + GO device are attributed to prevention of the multi-dimensional and random growth of conductive paths. Our XPS measurements infer that decrease in switching voltage upon adding BSA to enhanced oxygen vacancies. Detection of current ~nA in C-AFM investigation confirm the presence of filamentary switching. Present results will be helpful for future bio- RRAM devices, where detection of bio- molecules would be through memristor devices.

## Supplementary information

Supplementary info
