# Supplementary material for: Detection of bovine serum albumin using hybrid TiO2 + graphene oxide based Bio – resistive random access memory device
Source: Sci Rep. 2019 Nov 6;9:16141. doi: 10.1038/s41598-019-52522-w (PMC6834672; doi:10.1038/s41598-019-52522-w)
Supplement: Supplementary file 1 — Supplementary info [file 41598_2019_52522_MOESM1_ESM.pdf]

Supplementary Information

Detection of bovine serum albumin using hybrid TiO_2+graphene oxide based Bio – resistive random access memory device

Dwipak Prasad Sahu and S. Narayana Jammalamadaka*

Magnetic Materials and Device Physics Laboratory, Department of Physics, Indian Institute of Technology Hyderabad, Hyderabad, India – 502 285.

Figure S1(a) shows the UV Vis absorbance spectrum of the BSA protein with two absorption peaks at 239 nm and 280 nm. The former peak which corresponds to 239 nm has been believed as characteristic absorption peak for BSA and latter peak at 280 results from the aromatic amino acids. Convincingly, our results are in accordance with the results those have been obtained earlier [1]**.** Figure S1(b) depicts the variation of absorbance with wavelength for TiO_2, TB and TGB. Enhanced absorbance is evidenced upon adding BSA and GO to TiO_2. Essentially, presence of both the BSA and GO enhance the optical absorbance.

Attenuated total Reflectance-Fourier transformation infrared (ATR-FTIR) spectroscopy was recorded in order to see the presence of various bonds that are present in the BSA and TGB. Figure S1(c) shows the FTIR spectra for BSA and TGB. Essentially, FTIR is based on total internal reflection of IR radiation between the two interfaces by which we would be able to detect the protein- oxide interface interaction using the evanescent wave. During the measurement, first, the background spectrum was recorded and it was subtracted from the spectra of samples. The characteristic IR absorption bands for proteins are commonly referred to as the amide I, II, and III regions. The amide I, amide II and III region extend from 1600 to 1700 cm^{-1}, 1500 to 1600 cm^{-1} and 1200 to 1350 cm^{-1} respectively. [2] Two characteristic bands of BSA protein centered at 1641 and 1530 cm^{-1} are observed, which corresponds to symmetric stretching of C=O of amide I band and N−H bending vibration coupled to C=O, C = C stretching mode of amide II band respectively. [3] Amide III region has multiple smaller bands, but is

primarily composed of CH_2 scissoring motion. A peak at 1392 cm^{-1} was seen due to deprotonated form which is associated with C-O carboxylate stretch. [2] Two more broad peaks related to α-helix and β-sheet are observed at 1308 cm^{-1} and 1242 cm^{-1} respectively. [4] All the peaks are well indexed in Figure S1(c). The appearance of the peaks corresponding to amide I and amide II after conjugation of BSA to the TiO_2+GO surface hints the presence of protein in the final sample. Upon closer observation, it is evident that frequency for all the peaks shifted by a value of 5 cm^{-1}, which indicates that indeed there is an interaction at the surface of TiO_2+GO with BSA molecules. These changes indicate that the protein is unfolding and/or denaturing on the surface.

The morphological features of BSA molecules after encapsulation on TiO_2+GO surface were characterized by Field Electron Scanning Electron Microscopy at a voltage of 20 keV. Figure S1(d) shows SEM image of TiO_2/GO/BSA (TGB) film deposited on FTO substrate which reveals the spherical particle like morphology distributed on the surface of graphene oxide.

Fig. S2a represents the variation of absorbance spectra upon adding BSA protein to TiO_2. Starting from bottom of Fig. S2a, in the pure TiO_2 (T), we do see a single hump at around 210 nm. We do not see any other peak which corresponds to TiO_2. Upon adding BSA (B) in addition to parent T peak, we do see an evolution of BSA peak around 278 nm (this peak is specific for BSA). This indeed confirms that BSA is added to TiO_2 nanoparticles. Apart from that, peak at 210 nm modified drastically and the intensity enhances in addition to red shift of the peak. This indeed reveals that there is some interaction between BSA and TiO_2 which is due to unique structure of BSA protein. On top of that, peak broadening is observed with an increase in the concentration of BSA protein. The peak broadening can be explained based on the photochemical reaction of a small number of protein molecules which alters the chemical structure of the molecules. When such photo-activated molecules absorb light, the probability of these molecules tracing back to initial ground state is less, rather they shift to a new ground state whose absorption peak is different from the initial one, which leads to the broadening of

absorbance peak. Wang et. al has also reported the shift in maximum absorbance peak and peak broadening after conjugating human serum albumin to Ag nanoparticles. [5]

In addition to absorbance spectroscopy, we also performed static fluorescence spectroscopy to probe further in detection of BSA with different concentrations using TiO_2 nanoparticles. The steady-state fluorescence measurements were carried out with an excitation wavelength of 340 nm. The fluorescence intensity enhances as the concentration of BSA increases on TiO_2 nanoparticles as shown in Fig. S2b. Similar behavior has also been reported using gold nanorods upon adding BSA protein. [6]

Fig. S2c shows the standard curve to calculate the concentration of an unknown BSA sample. The left frame corresponds to absorbance related and the curve is drawn by considering the ratio of the change in UV absorbance peak intensity of BSA interacted sample (A) to that of non-interacted sample (A_0). On the right hand side, same figure indicates the average values of fluorescence intensity with respect to different concentrations of BSA, which helps to identify average fluorescence intensity of unknown BSA concentration. Fig. S2d infers shift in absorbance peak vs. BSA concentration, through which one can estimate BSA concentration if we know the value of wavelength at which absorbance maximum is known.

In order to elucidate the underlying conduction mechanism in TGB based memory device, we analyzed HRS and LRS states with different conduction models from which the transport property of carriers can be understood. In the HRS state, we find that the I-V characteristics curve follows Ohmic conduction behavior at lower bias region whereas the Schottky emission model fits best at higher voltage. Schottky mechanism has also been reported in many oxide and spinel ferrite memory device. [4,7] The expression relating current density with barrier height can be expressed as [7]

$$J_{SE} = A^{*}T^{2} \exp\left[\frac{-q(\phi_B - \sqrt{\frac{qE}{4\pi\varepsilon_0\varepsilon_i}})}{kT}\right]$$

J is the current density, A^* is the effective Richardson constant, q is the electronic charge, ε_0 is the permittivity of free space, ε_i is the dielectric constant, E is the electric field, and k is the Boltzmann's constant and $q\phi_B$ is Schottky barrier height. Figure S3a represents the ln I vs $V^{1/2}$ graph with a slope of 5 at higher bias field. Therefore, it is the energy barrier height at the interface due to which electrons are unable to overcome as electrons cannot obtain enough energy provided by the thermal activation. As a result, the filament formation remains incomplete and the resistance switched from LRS to HRS.

However, current conduction in LRS state is explained by trap controlled space charge limited current (SCLC) mechanism (Figure S3b in the Supporting Information). The double logarithmic scale of I-V curve of LRS state shows three regions, namely, Ohmic conduction, trap filled limited (TFL) behavior and transition from trap filled to Child's law. Initially, when the applied voltage across the device is small, the injected charge carrier density is smaller than the thermally generally generated free charge carrier density. The I-V curve fits to Ohm's law and the slope of the curve is 1. As the voltage increases, the injected carriers become dominant and a trap-filled limited current is observed in a region $V_{on} < V < V_{TFL}$, where V_{on} is defined as the transition voltage from ohmic to trap filled limited behavior and V_{TFL} is defined as the voltage required to fill all traps. When all the traps are filled, a jump in the current occurs at V_{TFL} and then the I-V follows Child's law at $V > V_{TFL}$. A space charge layer builds up in the switching media due to an excess number of free electrons. Eventually, the sample switches to LRS state at the set voltage. Thus, LRS mechanism can be related to the trapping of charge carriers via defects. The expression for Ohm's law, trap filled limited region and trap free region are given as follows:

$$J_{Ohm} = q\mu n_0 \frac{V}{d}$$

$$J_{TFL} = \frac{9}{8}\mu\varepsilon\theta\frac{V^2}{d^3}$$

and

$$J_{Child} = \frac{9}{8}\mu\varepsilon\frac{V^2}{d^3}$$

Where n_0 is the concentration of free charge carriers, μ is electron mobility, V is applied voltage, d is the thickness of the film, ε is the dielectric constant, θ is the ratio of the free charge carrier density to total carrier density.

References:

1. H. Xu, N. Yao, H. Xu, T. Wang, G. Li, Z. Li, *Int. J. Mol. Sci.* **2013**, 14, 14185.
2. B. E. Givens, Z. Xu, J. Fiegel, V. H. Grassian, *J. Colloid Interface Sci.* **2017**, 493, 334.
3. Z. Xu, V. H. Grassian, *J. Phy. Chem. C* **2017**, 121, 21763.
4. W. Zhu, T. P. Chen, Y. Liu, S. Fung, *J. Appl. Phys.* **2012**, *112*, 063706.
5. Wang, Y., and Yongnian Ni. Analyst 139, 416-424 (2014)
6. Shajari, D., Bahari, A., Gill, P. Colloids and Surfaces A: Physicochemical and Engineering Aspects 543, 118-125, (2018).
7. W. Hu , N. Qin , G. Wu , Y. Lin , S. Li , D. Bao , *J. Am. Chem. Soc*. **2012**, *134*, 14658 .

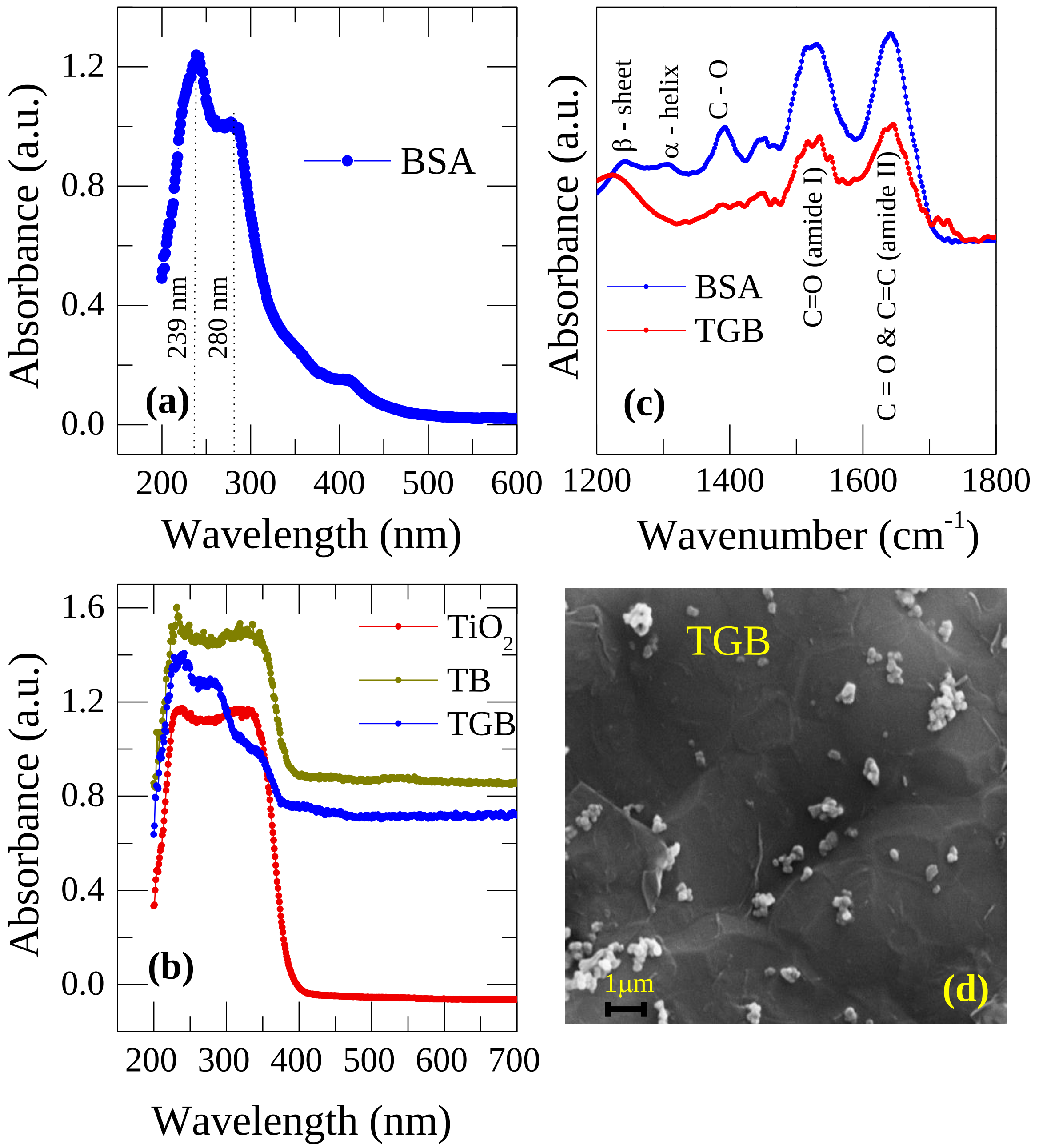

Figure S1: (a) UV Vis absorbance spectrum of pure BSA protein with a strong absorption peaks at 239 nm reflects the backbone of BSA, while the peak at 280 nm resulted from the aromatic amino acids. **(b)** Absorbance spectra of TiO_2, TiO_2-BSA(TB), TiO_2-GO-BSA(TGB). UV spectrum of TGB indicates attachment of BSA protein on to TGB composite. **(c)** ATR-FTIR spectra of pure BSA and TGB. The characteristic IR absorption bands for proteins are associated with the amide I, II, and III regions. The amide I, II, and III regions extend from 1600-1700 cm^{-1}, 1500-1600 cm^{-1} and 1200-1350 cm^{-1}, respectively. Two characteristic peaks of protein were remained after conjugation to the TiO_2-GO surface indicating the presence of protein in the final sample. **(d)** FE-SEM image of BSA molecules after encapsulation on TiO_2-GO surface.

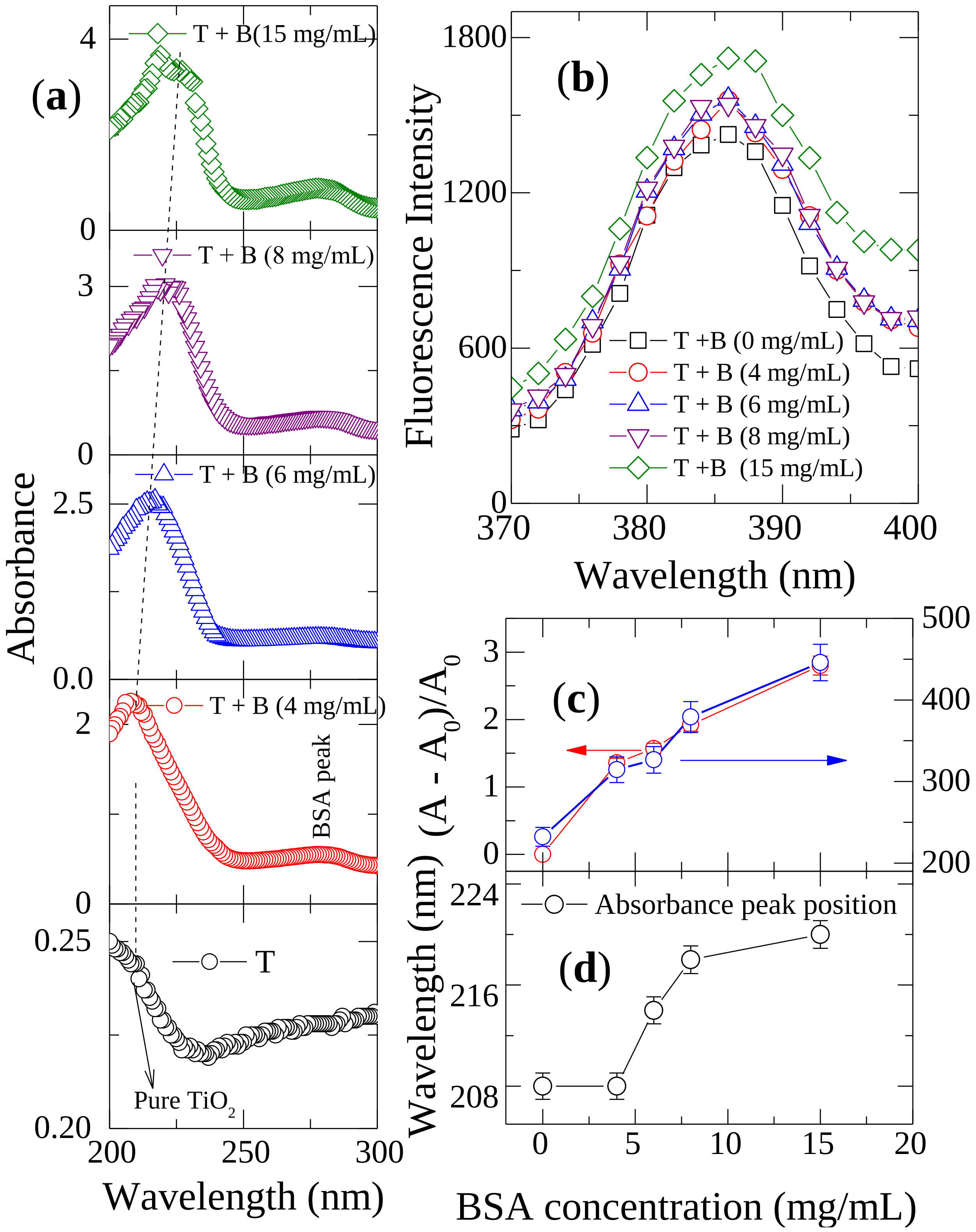

Fig. S2: a) UV – Vis spectra variation upon adding different concentrations of BSA to TiO_2 NPs. B) Flouorescence spectra for different concentrations of BSA. (c) variation of UV absorbance peak intensity of BSA interacted sample (A) to that of non-interacted sample (A_0) and average fluorescence intensity with respect to the BSA concentration. (d) wavelength at which absorption maximum observed vs. BSA concentration

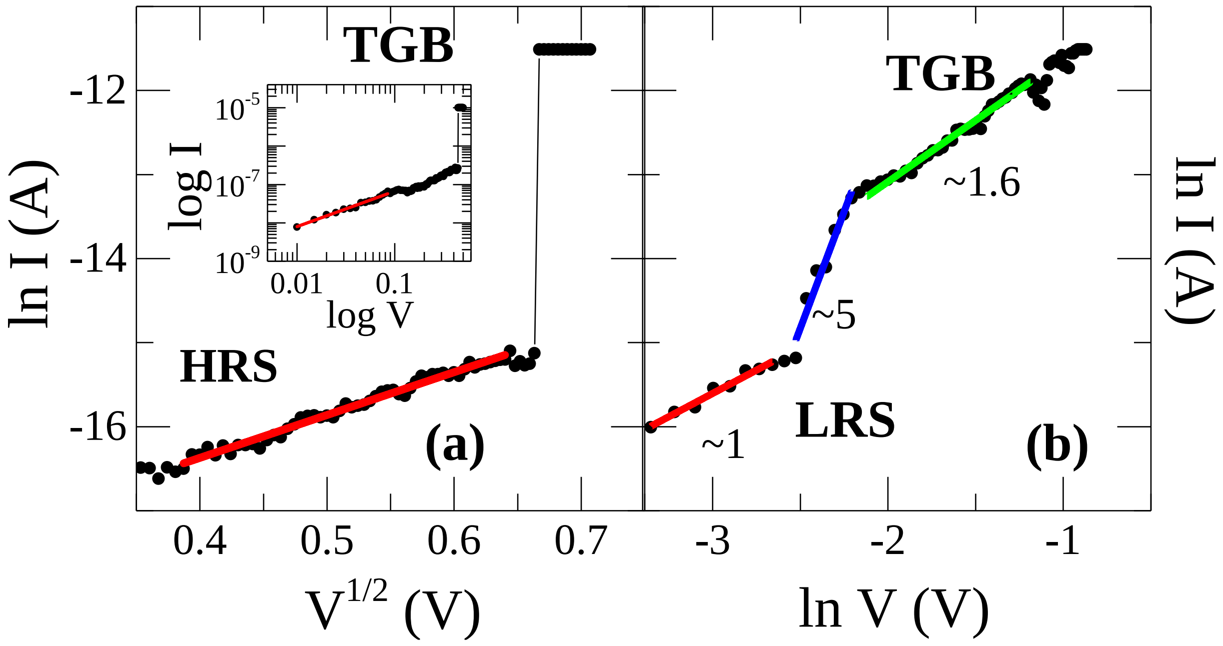

Figure S3: Fitting results of the HRS and LRS for the I-V curves in the positive voltage region of the Ag/BSA/GO/TiO_2/FTO (TGB) based device. **(a)** HRS fits to Schottky emission **(b)** Trap controlled space charge limited current (SCLC) mechanism is followed by LRS plotted on double logarithmic scale.

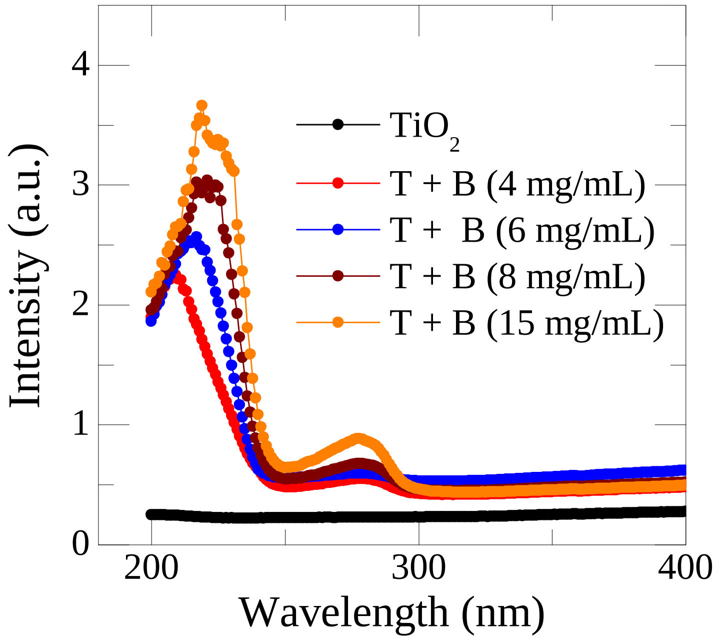

Figure S4: Full range of absorption spectra for various concentrations of BSA.
